# Health Natural Language Processing: Methodology Development and Applications

**DOI:** 10.2196/23898

**Published:** 2021-10-21

**Authors:** Tianyong Hao, Zhengxing Huang, Likeng Liang, Heng Weng, Buzhou Tang

**Affiliations:** 1 School of Computer Science South China Normal University Guangzhou China; 2 College of Computer Science and Technology Zhejiang University Guangzhou China; 3 The Second Affiliated Hospital Guangzhou University of Chinese Medicine Guangzhou China; 4 School of Computer Science and Technology Harbin Institute of Technology Shenzhen Shenzhen China

**Keywords:** health care, unstructured text, natural language processing, methodology, application

## Abstract

With the rapid growth of information technology, the necessity for processing substantial amounts of health data using advanced information technologies is increasing. A large amount of valuable data exists in natural text such as diagnosis text, discharge summaries, online health discussions, and eligibility criteria of clinical trials. Health natural language processing, as an interdisciplinary field of natural language processing and health care, plays a substantial role in a wide scope of both methodology development and applications. This editorial shares the most recent methodology innovations of health natural language processing and applications in the medical domain published in this JMIR Medical Informatics special theme issue entitled "Health Natural Language Processing: Methodology Development and Applications".

## Introduction

Text data in an unstructured format widely exists in the medical domain, such as diagnosis records, operation records, discharge summaries, eligibility criteria of clinical trials, social media comments, online health discussions, and medical publications. Natural language processing (NLP) is a field of computer science, artificial intelligence, and computational linguistics concerned with the interactions between computers and human (natural) language texts. NLP aims to provide computer programs with the ability to process and understand unstructured text data. In the health arena, NLP techniques have been shown to be useful in dealing with information overload in the health and medical domain (eg, aggregation and summarization of patient notes, treatment analysis, information extraction and retrieval from massive discharge summaries, and semantic understanding of patient queries) [1]. NLP may also be applied for assisting medical decision-making by automatically analyzing the commonalities and differences of a large amount of text data and recommending appropriate actions on behalf of domain experts [2].

Health NLP, as an interdisciplinary field of NLP and health care, focuses on the methodology development of NLP and its applications in health care. It facilitates the analysis of the commonalities and differences of large amounts of text data and recommends appropriate actions on behalf of domain experts to assist medical decision-making. In general, it plays an essential role in processing various types of health text data and supports health applications to improve health care efficiency and efficacy.

With the increasing attention on this research field, there are more and more developments related to health NLP. Velupillai et al [3] shared the recent advances of health NLP in support of semantic analysis, covering the development of efficient methods for health corpus annotation/deidentification and the leverage of NLP for clinical utility including NLP infrastructure for a clinical use case. Kalyan and Sangeetha [4] investigated the embeddings in health NLP for text representation in deep learning–based NLP tasks in clinical domains. The National NLP Clinical Challenges/Open Health Natural Language Processing (OHNLP) Competition [5] is held for family history extraction from synthetic clinical narratives using NLP. A number of health NLP tools and systems were also developed. For example, OHNLP released a catalog of clinical NLP software and provides interfaces to simplify the interaction of NLP systems [6]. Typically, Apache cTAKES [7], as an NLP system for extraction of information from electronic medical record clinical free text, aimed to integrate best-of-breed annotators, providing a world-class NLP system for accessing clinical information within the free text.

## Methods

Health NLP covers a wide scope of methodology research including topics about methodology research such as NLP models for medical or social web data (eg, literature, EHRs, clinical trials, and social media about health care) processing; health information retrieval and extraction; NLP-assisted health information aggregation, abstraction, and summarization; machine learning–based text mining methods for health care; health care knowledge representation and reasoning; heath text corpora construction and annotation; and medical ontology and health knowledge graphs construction.

With respect to the application of NLP methods to the health care domain, health NLP contains NLP techniques for medicine personalization; question answering technologies for health applications; novel tools for medical, clinical, or social web data interpretation and visualization; innovative NLP systems for mobile environments for health care applications; NLP for clinical decision support and informatics; and applications of advanced NLP methods in clinical practice.

We reviewed 10 published articles in the JMIR Medical Informatics theme issue Health Natural Language Processing: Methodology Development and Applications to share the recent developments of the studies, from methodology research to applications.

## Results

### Medical Information Extraction

Medical information extraction is a key technology that supports the development of medical informatics. Zhang et al [8] developed a new Chinese electronic medical record (EMR) data set with six types of entities and proposed a multilevel representation learning model based on Bidirectional Encoder Representation from Transformers (BERT) for Chinese medical entity recognition. The experiments on the Chinese EMR data set and China Conference on Knowledge Graph and Semantic Computing 2018 benchmark data set showed that the proposed method outperformed state-of-the-art methods. Automatic relation extraction between chemicals and diseases plays an important role in biomedical text mining. Wang et al [9] proposed an end-to-end neural network based on a graph convolutional network and multi-head attention. To improve the performance, a document-level dependency graph was constructed to capture dependency syntactic information across sentences. The graph was applied to capture the feature representation of a document-level dependency graph, while the multi-head attention mechanism was used to learn relative context features from different semantic subspaces. The experiment results showed that the method achieved the best F-score, which was superior to state-of-the-art methods. The graph convolutional network model was effectively used for dependency information across sentences to improve the performance of intersentence chemical-disease extraction. Targeted at extracting the interactions between chemicals and proteins from the biomedical literature, Wang et al [10] proposed effectively encoding syntactic information from long text. The method leveraged graph convolutional networks to capture sequential information and long-range syntactic relations between words by using the dependency structure of input sentences. The evaluation of the ChemProt corpus showed that the model achieved an F-score of 65.17%, which was 1.07% higher than that of the state-of-the-art system. The study indicated that the graph neural network–based model could better capture the semantic and syntactic information of the biomedical literature sentence. Temporal information frequently exists in the representation of the disease progress, prescription, medication, surgery progress, or discharge summary in narrative clinical text. The extraction and normalization of temporal expressions can positively boost the analysis and understanding of narrative clinical texts to promote clinical research and practice. Pan et al [11] proposed a rule-based and pattern learning–based model for extracting and normalizing temporal expressions from Chinese narrative clinical text. The model consisted of three stages: extraction, classification, and normalization. Based on a set of narrative clinical texts in Chinese containing 1459 discharge summaries of a domestic Grade-A Class 3 hospital, the performance of the model achieved the performance compared with baseline methods. The research of medical information extraction still has the challenges of insufficient training data size, complex domain terminology, a large proportion of noise data, and significant inconsistency among various data types.

### Heath Knowledge Graph and Its Applications

Targeted at knowledge graph embedding for semantic representation of entities and relations, the challenge of how to learn probability values of triplets into representation vectors was addressed. Li et al [12] constructed a mapping function between score value and probability, and introduced probability-based loss of triplets into original margin-based loss function. Compared with state-of-the-art TransX algorithms, the proposed model performed better in all evaluation indicators. Checking whether the medication is clinically reasonable with respect to the diagnosis is the key to fraud, waste, and abuse detection, which is a significant yet challenging problem in the health insurance industry. Sun et al [13] built an automatic method to identify the clinically suspected claims for fraud, waste, and abuse detection by using a medical knowledge graph. A deep learning–based method was applied to extract the entities and relationships from knowledge sources, and a multilevel similarity matching method was developed for entity linking. From 185,796 drug labels from the China Food and Drug Administration, a medical knowledge graph containing 1,616,549 nodes and 5,963,444 edges was constructed for identifying fraud, waste, and abuse suspects. The research of health knowledge graphs still has the challenges of complex text representation, low extract performance, and limited knowledge graph size.

### NLP Methods for Health Text Mining

Traditional Chinese medicine (TCM) has been shown to be an efficient mode to manage advanced lung cancer, and accurate syndrome differentiation is crucial to treatment. Liu et al [14] established five deep learning–based TCM diagnostic models to imitate lung cancer syndrome differentiation. The models used unstructured medical records as inputs to capitalize on data collected for practical TCM treatment cases by lung cancer experts. The experiment result showed the F1-score of the recurrent convolutional neural network model improved over models without data augment. The text-hierarchical attention network model achieved the highest F1-score. Medical records could be used more productively by constructing end-to-end models to facilitate lung cancer. The classification of clinical trial eligibility criteria texts is a fundamental and critical step in clinical target population recruitment. Zeng et al [15] proposed an ensemble learning method that integrates the current cutting-edge deep learning models BERT, Enhanced Language Representation with Informative Entities, XLNet, and RoBERT. Through a model ensemble in two layers, the study trained a model and compared it with a list of baseline deep learning models on a publicly available standard data set. The results demonstrated that the proposed ensemble learning method outperformed a list of baseline methods. The research of NLP methods still heavily relies on the advancement of machine learning models.

### Advanced Applications

Deidentification of clinical records, as an application, is a critical step in the use of electronic health records for academic research. Zhao et al [16] investigated the usefulness of rule-based learners in a hybrid deidentification system. A data-driven rule learner named transformation-based error-driven learning was integrated into a hybrid system. Based on the widely used Informatics for Integrating Biology and the Bedside deidentification data set, the learner could offer high performance with generated rules. After integrating the learner into an ensemble framework, the performance achieved the best among the community. The rule-based method thus could offer an effective contribution to the current ensemble learning approach for the deidentification of clinical records as a typical application in medical informatics. An artificial intelligence–based assistive diagnostic system is designed to diagnose multiple types of diseases that are common in TCM based on patients’ electronic health record notes. Zhang et al [17] developed a method to simultaneously diagnose the disease and produce a list of corresponding syndromes. NLP techniques using a recurrent neural network model were applied to process unstructured electronic health record notes to extract clinical information such as signs and symptoms that were represented by named entities. A total of 22,984 electronic health records from Guanganmen Hospital of the China Academy of Chinese Medical Sciences were collected and applied to the diagnostic system. From the evaluation, 187 commonly known TCM diseases could be diagnosed, and a wider range of TCM disease types was expected to be diagnosed. The applications of NLP methods tend to be more and more widespread in the health care domain. However, the challenges, including the security of data, the actual needs from clinicians, the validation of results, and user convenience, still need to be solved in the future.

## Conclusion

Health NLP draws more and more attention for its essential role in a wide scope of both methodology development and applications. This editorial shares the most recent methodology research of health NLP and its applications in health care by reviewing 10 newly published articles on the JMIR Medical Informatics theme issue Health Natural Language Processing: Methodology Development and Applications. The research indicates recent focuses on medical information extraction (entity, relation, temporal, and interaction extraction), knowledge graph construction and use, methods for clinical decision support and informatics, and NLP systems for health care applications in practice.

## Abbreviations

BERT: Bidirectional Encoder Representation from Transformers
NLP: Natural Language Processing
OHNLP: Open Health Natural Language Processing
TCM: Traditional Chinese Medicine
